# Similar Prognoses for Invasive Micropapillary Breast Carcinoma and Pure Invasive Ductal Carcinoma: A Retrospectively Matched Cohort Study in China

**DOI:** 10.1371/journal.pone.0106564

**Published:** 2014-09-04

**Authors:** Yin Liu, Xiaoyan Huang, Rui Bi, Wentao Yang, Zhimin Shao

**Affiliations:** 1 Department of Breast Surgery, Fudan University Shanghai Cancer Center/Cancer Institute, Shanghai, People’s Republic of China; 2 Department of Oncology, Shanghai Medical College, Fudan University, Shanghai, People’s Republic of China; 3 Department of Pathology, Fudan University Shanghai Cancer Center/Cancer Institute, Shanghai, People’s Republic of China; 4 Institutes of Biomedical Science, Fudan University, Shanghai, People’s Republic of China; University of Campinas, Brazil

## Abstract

**Purpose:**

Invasive micropapillary breast carcinoma (IMPC) is a rare pathological finding. Few studies have compared IMPC with invasive ductal breast carcinoma (IDC) according to matched nodal status and age. To better illustrate the difference between IMPC and IDC prognoses, we conducted this cohort study.

**Methods:**

51 mixed or pure IMPC patients and 102 pure IDC patients were matched for nodal status and age. Clinical and biological features as well as disease-free survival (DFS) were compared between groups.

**Results:**

More than one-half of IMPC consisted of mostly or exclusively IMPC component (meaning greater than 75%) and these tumors significantly correlated with a higher histologic grade (*P* = 0.016) and LVI positivity (*P* = 0.036) compared with mixed IMPC. IMPC displayed a significantly higher rate of estrogen receptor (ER) positivity and lymphovascular invasion (LVI) compared to matched IDC. Women diagnosed with IMPC had a slight, but not significant, reduced frequency for recurrence and metastasis compared to women with IDC (15.7% vs. 21.6%, *P* = 0.518). In the subgroup analysis, IMPC patients demonstrated significantly reduced survival (*P* = 0.018) compared to IDC patients in the T_1_N_2–3_ subpopulation, whereas IDC patients demonstrated significantly increased recurrence and metastasis (*P* = 0.024) compared to IMPC patients in the T_2_N_2–3_ subgroup. No difference was observed in patients with 3 or less positive lymph nodes (LNs).

**Conclusion:**

Although no difference in DFS was observed between IMPC and LN-matched IDC patients, IMPC patients demonstrated a significantly poorer outcome compared to IDC patients with smaller tumors and 4 or more positive LNs. The opposite results were observed in larger tumors and patients with 4 or more positive LNs. Therefore, we might advise more proactive treatment for IMPC patients with a smaller tumor size and extensive LN involvement. Furthermore, correlative IMPC studies should focus on this subset of patients to elucidate the genetic and/or biologic differences that contribute to metastatic potential.

## Introduction

**Figure 1:**
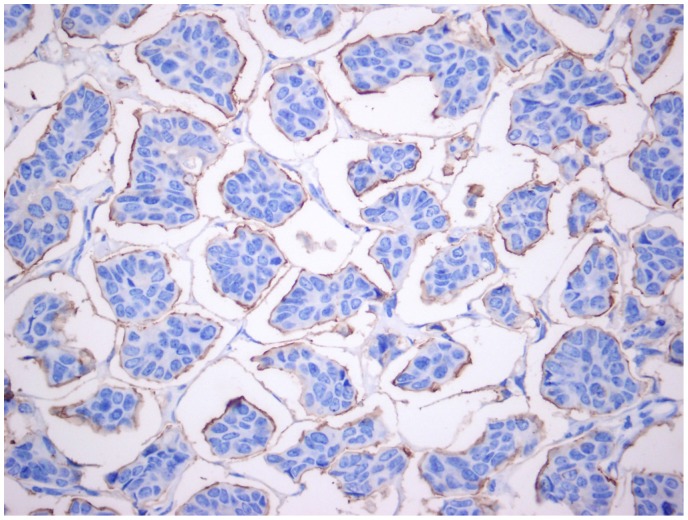
Positive stain for EMA antigen on the peripheral cell membranes is suggestive of “inside-out” morphology.

Invasive micropapillary carcinoma (IMPC) of the breast is a rare variant of invasive breast cancer that is more likely to be associated with nodal metastasis and lymphatic invasion [1], [2], [3]. The incidence of IMPC ranges from 3 to 6% [4] of all primary breast cancers. Compared to invasive ductal breast carcinoma (IDC), IMPC is characterized by cells arranged in pseudopapillary structures and surrounded by clear empty spaces lined by delicate strands of fibrocollagenous stroma [5], [6] [shown in **Figure** 1].

Although it is widely accepted that IMPC has an unfavorable prognosis, a debate exists as to whether this prognosis is attributed to increased nodal stage on initial diagnosis or other intrinsic IMPC biological behaviors. To better understand the difference between IMPC and IDC prognoses, we conducted this cohort study.

## Materials and Methods

### Ethics Statement

The study was conducted according to the principles expressed in the Declaration of Helsinki and approved by the institutional review board of Shanghai Cancer Center, Fudan University. All the patients enrolled in this study have signed the informed consent voluntarily.

### Controls, matching criteria and follow-up

The cases and controls were matched at a ratio of 2∶1 in the IMPC group.

We retrospectively reviewed consecutive data from 51 operable IMPC patients who were histopathologically ascertained and treated in the Department of Breast Surgery at the Cancer Hospital/Institute of Fudan University (Shanghai, China) from August 1, 2005 to March 1, 2008. During this time, a total of 1,951 patients received operations at this institution. In total, 102 pure IDC patients treated during the same time and at the same institute were matched according to lymph node (LN) status and age at diagnosis as controls. We observed the cohort until the median follow-up duration was greater than 5 years.

According to the inclusion criteria, all cases were confirmed as females with IMPC or pure IDC without special types and lacking distant metastasis at the initial diagnosis. All patients received a complete physical examination, bilateral mammography, chest radioscopy, ECG and ultrasonography of the breast, axillary fossa, abdomen and pelvis. All patients at risk for relapse received adjuvant chemotherapy composed of different regimens according to the standards used at the time of surgery followed by radiotherapy (if required) and/or endocrine therapy (if required).

The study was approved by the Scientific and Ethical Committee of the Cancer Hospital/Institute of Fudan University, Shanghai, China. All hospitalized patients agreed to the use of their tumor samples and clinical information without divulging personal information at future investigations performed many years after the initial diagnosis.

Follow-up data were collected annually regarding disease recurrence, metastasis, second primary carcinoma and death from a variety of factors. Patient information was acquired by telephone contact and routine clinical follow-up.

### Diagnostic criteria for IMPC

Hematoxylin and eosin (H&E)-stained FFPE sections from the database were reassessed by 2 senior pathologists (BR and YWT). Divergent opinions were discussed until a consensus was reached. Slides stained with H&E from all blocks for each tumor were reviewed by 2 senior breast pathologists (BR and YWT) to verify the presence of IMPC according to the morphologic criteria described in the WHO histologic classification of breast tumors [7]. The IMPC component for each tumor was graded according to the Elston and Ellis [8] grading system as follows:

- grade I, no significant nuclear atypia and a mitotic count of 1 or less per 10 high-power fields (HPF);
- grade II, moderate nuclear atypia and a mitotic count of 2 to 3 per 10 HPF;
- grade III, marked nuclear atypia and a mitotic count of greater than 4 or more per 10 HPF.

We used the same grading system [8] to evaluate the IDC grade. For invasive micropapillary carcinomas with some IDC component, use of the higher grade is widely accepted. The IMPC specimens were classified into 3 groups according to the amount of IMPC present in each tumor: tumors with less than 25%, tumors with 25% to 74% and tumors with greater than 75% IMPC. Lymphovascular invasion (LVI) was assessed as follows: negative, no IMPC cells in lymph vessels, single lymphatic vessel dilated by IMPC cells and multiple lymphatic vessels dilated by IMPC cells.

### IHC evaluation

ER, PR and HER2 status was determined on representative paraffin sections from each tumor using immunohistochemical (IHC) staining. The assessment was performed 1 week after patient surgery. The ER (M7047, clone 1D5, Dako, Produktionsvej, Glostrup, Denmark) and PR (M3569, clone PgR 636, Dako) antibodies were purchased from Dako, and these antibodies were evaluated using an avidin-biotin-peroxidase complex (ABC) assay. ER and PR expression was considered positive in breast cancer cells if the number of positive nuclei was >1%. Cytoplasmic staining was not evaluated. We performed semi-quantitative ER assessment defined as follows: score 0, <1% positive nuclei; score 1+, less than 10% positive nuclei; score 2+, less than 50% positive nuclei and score 3+, greater than 50% positive nuclei. Overexpression of the HER2 protein was evaluated using a monoclonal antibody (Dako, Clone PN2A 1∶400) and a peroxidase-antiperoxidase (PAP) technique. The results are presented on a qualitative scale from 0 to 3+ according to the criteria of the HercepTest [9]. The HER2 membrane staining intensity and pattern were evaluated using the 0 to 3+ scale. Scores of 0 and 1+ (weak immunostaining in less than 10% of tumor cells) were defined as negative, 2+ (complete membrane staining in at least 10%, but less than 30%, of tumor cells) as equivocal and 3+ (uniformly intense membrane staining in at least 30% of tumor cells) as positive. In our study, only the 3+ IHC classification of HER2 status was defined HER2 positive. The pathological and IHC studies were performed using an Olympus light microscope with x10 and x40 magnifications by the 2 above-noted independent pathologists in the Department of Pathology of the Cancer Hospital/Center at Fudan University.

### Statistical analysis

The primary end point of the study was disease-free survival (DFS), which was defined as the time from surgery to local recurrence, relapse or metastasis. Second primary breast cancers were not included in the DFS calculations. To distinguish the clinicopathological features, we used Fisher’s exact test and the Pearson chi-square test. Survival curves were generated using Kaplan-Meier methods, and we used the log-rank test to determine the statistical significance of comparative survival for different tumor characteristics. Multivariate and univariate analysis using bootstrap resampling of the Cox proportional hazard ratio regression model was performed. Matching was performed according to the 2 variables mentioned above using SAS 8.2 (SAS, NC, USA) initially, and ages were matched according to age group (30–39, 40–49, 50–59, 60–69, 70–79 and 80–89). For all statistical tests, statistical significance was limited to P<0.05, and all P values were 2-sided. The program SPSS 19.0 (IBM SPSS Statistics 19) was used for the analysis.

## Results

### Characteristics of patients

**Figure 2:**
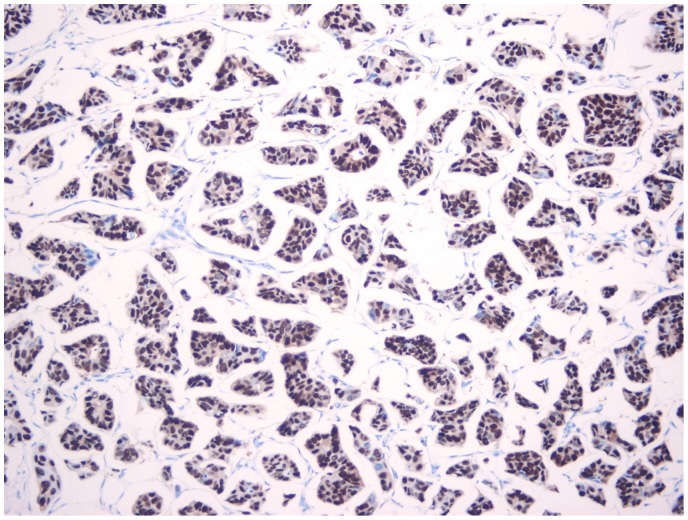
Diffuse positive stain for ER in IMPC component.

The IPMC (pure or mixed with IDC) group included 51 patients ranging in age from 26 to 83 years, and the median age was 51 years. The IDC group included 102 patients ranging in age from 28 to 83 years, and the median age was 52 years. The average tumor size in the IMPC and IDC groups was 2.72 cm and 2.59 cm, respectively (*P* = 0.249). The IMPC group displayed a significantly increased rate (*P* = 0.005) of LVI compared to the IDC group, and most of the diagnoses were multi-LVI. In addition, multifocal lesions presented more often (*P* = 0.009) in the IMPC group than the IDC group. Moreover, multifocal lesions significantly correlated with HER2 positivity (*P* = 0.011). Interestingly, patients in the IMPC group demonstrated a significantly higher rate (*P* = 0.037) of ER positivity compared to their IDC counterparts, although we cannot explain this observation [**Figure 2**]. The characteristics of 153 IMPC and IDC patients are shown in **Table** 1.

**Table 1:** Baseline characteristics and treatment patterns for all patients.

characteristics	IMPC^1^		IDC^2^		*P-value* 3 *
	(n = 51)		(n = 102)		
	n	%	n	%	
**Average age(y)**	50.94±10.9		51.53±11.6		*p* = 0.764
**Median age(y)**	51		52		
**Range of age(y)**	26–82		28–83		
< = 50 years	23	45.1	46	45.1	*p = 1.000*
>50 years	28	54.9	56	54.9	
**gender**	female		female		
**Nodal status**					
N0	15	29.4	29	28.43	*p = 0.950*
N1	10	19.61	24	23.53	
N2	15	29.41	27	26.47	
N3	11	21.57	22	21.57	
**Tumor size**					
**Average size(cm)**	2.72±0.20		2.59±0.11		
T1	22	43.14	38	37.25	*p = 0.333*
T2	24	47.06	60	58.82	
T3	3	5.88	3	2.94	
Tx	2	3.92	1	0.98	
**Grade** 4 **					
I	2	3.92	2	1.96	
II	24	47.06	63	60.78	
III	25	49.02	37	36.27	
**Lymphovascular-invasion**					
yes	27	52.94	30	29.41	*p = 0.005*
no	24	47.06	72	70.59	
**Multifocality**					
yes	10	19.61	6	5.88	*p = 0.009*
no	41	80.39	96	94.12	
**ER** 5					
positive	43	84.31	70	68.63	*p = 0.037*
negative	8	15.69	32	31.37	
**PR** 6					
positive	37	72.5	61	59.8	*p = 0.121*
negative	14	27.4	41	40.2	
**HER2/neu** 7					
negative	42	84.31	82	80.4	*p = 0.261*
positive	8	15.69	20	19.6	
**Surgery**					
Auchincloss	46	72.55	93	73.53	*p = 1.000*
BCS^8^	5	7.84	9	6.86	
**Chemotherapy**					
**Anthracyclines**					
yes	47	92.16	81	79.41	*p = 0.044*
no	4	7.84	21	20.59	
**Taxanes**					
yes	21	41.18	31	30.39	*p = 0.184*
no	30	58.82	71	69.61	
**Both taxanes and anthracyclines**					
yes	21	41.18	29	28.43	*p = 0.113*
no	30	58.82	73	71.57	
**Endocrine therapy**					
yes	45	88.24	73	71.57	*p = 0.021*
no	6	11.76	29	28.43	

1 **IMPC:** invasive micropapillary breast carcinoma;

2 **IDC:** invasive ductal carcinoma not otherwise specified;

3 *All the chi-square analysis has been tested by bootstrapping;

4 **Grading in IMPC and IDC was slightly different because IMPC was characterized by cells arranged in pseudopapillary structures, without typical ductal structure;

5 **ER:** estrogen receptor status;

6 **PR:** progesterone receptor status;

7 **HER2/neu:** human epidermal growth factor receptor 2 status;

8 **BCS:** breast-conservative surgery.

### Treatment

Forty-six (90.2%) IMPC patients received modified radical mastectomy, whereas 95 (93.1%) of the IDC patients received this surgery. Breast-conserving surgery (BCS) was performed on the remaining patients in each group. In both groups, re-excision was performed if the margin was not devoid of carcinoma during the BCS procedure. All patients with positive node metastasis received axillary LN (ALN) dissection.

Patients from both groups treated with BCS who demonstrated a primary tumor larger than 5 cm and/or 4 or more positive ALNs received postoperative radiation therapy (RT) on the breast and chest wall. In addition, all patients treated with BCS also received an electron boost to the tumor bed.

Systemic chemotherapy was administered to 131 patients (85.6%) according to the standard at that time. We observed a significantly higher proportion (92.2%, *P* = 0.044) of patients in the IMPC group receiving a regimen containing anthracycline compared to the IDC group (79.4%). Fifty-two (33.9%) patients received a chemotherapy regimen containing paclitaxel, but no difference was observed between the 2 groups. Hormone therapy was administered to 108 patients (70.6%) who were positive for hormone receptors. Of these patients, 45 (88.24%) were IMPC patients and 73 (74.5%) were IDC patients. HER2 targeted therapy was not routinely used in the period of the systemic treatment, so no trastuzumab was used in our study.

### The IMPC component, LVI and other pathological features

**Figure 3:**
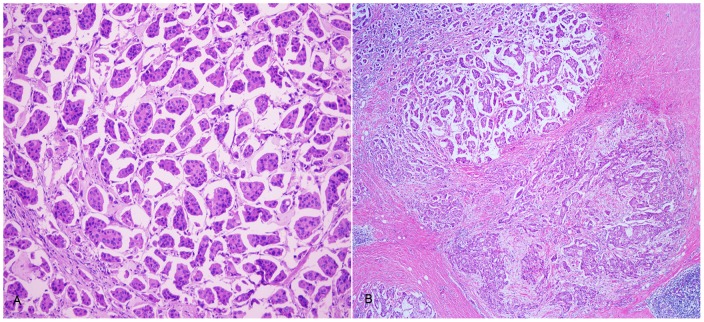
Pure and mixed invasive micropapillary carcinoma. Pure invasive micropapillary carcinoma, ×100 original magnification (A); Mixed carcinoma with IMPC (left up area) and invasive ductal carcinoma, not otherwise specified (right low area), ×40 original magnification (B).

The 51 cases of breast carcinoma with an IMPC component were classified into 3 groups according to the amount of IMPC present (as described in the IMPC diagnostic criteria). The pathological features of the IMPC tumors were shown in **Table** 2. More than one-half of tumors in this group consisted of mostly or exclusively IMPC (meaning greater than 75%) [**Figure 3A, B**], and these tumors significantly correlated with a higher histologic grade (*P* = 0.016) and LVI positivity (*P* = 0.036). As expected, these tumors exhibited increased LN metastasis, although this increase was not statistically significant.

**Table 2:** The pathologic characteristics between IMPC patients with and without recurrence.

	IMPC^1^ with recurrence	IMPC without recurrence
**Total number**	8	43
**LVI** 2		
Without LVI	1(12.5%)	17(39.5%)
Single LVI	0(0%)	8(18.6%)
Multiple LVI	7(87.5%)	18(41.9%)
**Proportion of IMPC**		
<25%	3(37.5%)	5(11.6%)
25–75%	3(37.5%)	14(26.4%)
>75%	2(25%)	24(55.8%)
**Nuclear Grade**		
G1^3^	0(0%)	2(4.7%)
G2^4^	4(50%)	20(46.5%)
G3^5^	4(50%)	21(48.8%)

1 **IMPC:** invasive micropapillary carcinoma;

2 **LVI:** Lymphovascular invasion;

3 **G1:** Grade 1;

4 **G2**: Grade 2;

5 **G3:** Grade 3.

**Figure 4:**
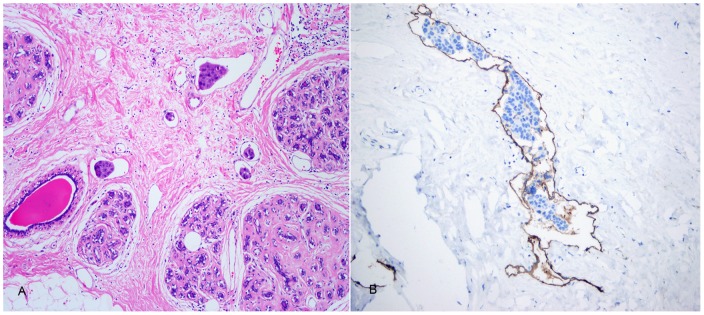
IMPC with lymphovascular invasion. Extensive lymphovascular invasion (LVI), ×100 original magnification (A); The endoepithelial cells were positive for D2-40 by immunohistochemical stain, ×100 original magnification (B).

Approximately one-half of the IMPC patients were diagnosed with multi-LVI (**shown in** **Figure 4A, B**), and these patients exhibited a significantly higher proportion of LN metastases (*P*<0.001) and multifocal lesions (*P* = 0.019).

### Survival of subgroups according to tumor size and node status

**Figure 5:**
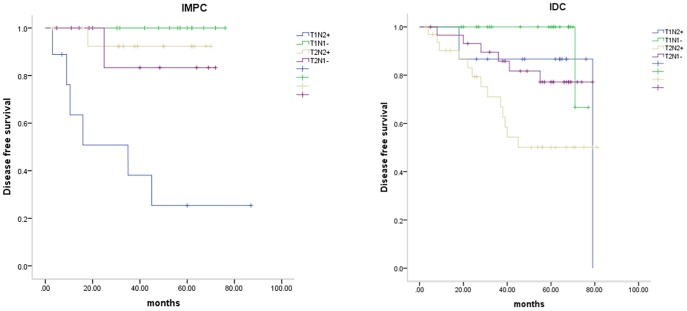
Cumulative disease free survival rate curve according to tumor size and lymph node status in invasive micropapillary carcinoma (IMPC) (*P*<.001) and invasive ductal carcinoma (IDC) (*P* = .009).

Cox proportional hazard model that included group, stage and group*stage showed interaction between stage and group (P = 0.032). Hence we classified IMPC and IDC patients into the following 4 categories according to tumor size and node status: T_1_N_0–1_, T_1_N_2–3_, T_2–3_N_0–1_ and T_2–3_N_2–3_. The clinicopathological features of the tumors from each group are summarized in **Table** 3. Univariate analysis using the Kaplan-Meier method indicated that the T_1_N_2–3_ subgroup was associated with poor prognosis in the IMPC group, whereas the T_2–3_N_2–3_ subgroup displayed the highest rate of recurrence and metastasis in the IDC group. The cumulative disease-free-survival rate curves according to tumor size and lymph node status were shown in **Figure 5**. Then additional univariate Cox regression by bootstrapping was tested in this survival comparison. We found out that T_1_N_2–3_ subgroup was still associated with poor prognosis in the IMPC group (*P* = 0.001, RR 20.082, 95% CI 4.38–59874.14), whereas the T_2–3_N_2–3_ subgroup displayed the highest rate of recurrence and metastasis in the IDC group (*P* = 0.003, RR 3.531, 95% CI 1.53–9.45).

**Table 3:** Characteristics of patients in defined subgroups.

Characteristics	T_1_N_2–3_		T_1_N_0–1_		T_2_N_2–3_		T_2_N_0–1_	
	IDC^1^	IMPC^2^	IDC	IMPC	IDC	IMPC	IDC	IMPC
**toal**	15	9	23	14	33	17	30	11
**recurrence**	3	6	1	0	13	1	6	1
**ER** 3								
positive	13	7	18	14	21	14	17	9
negative	2	2	5	0	12	3	13	2
**PR** 4								
positive	10	6	15	12	20	11	15	9
negative	5	3	8	2	13	6	15	2
**Her2/neu** 5								
positive	1	1	2	1	1	2	3	1
negative	14	8	21	13	32	15	27	10
**Grade**								
1	0	0	0	0	1	2	0	0
2	11	5	13	10	18	4	21	5
3	4	4	10	4	14	11	9	6
**LVI** 6								
positive	6	6	5	7	14	13	5	2
negative	9	3	18	7	19	4	25	9
**Multifocality**								
positive	0	5	2	3	3	3	1	0
negative	15	4	21	11	30	14	29	11
**Anthracyclines**								
use	12	9	16	13	28	17	24	8
no use	3	0	7	1	5	0	6	3
**Taxanes**								
use	5	5	2	2	18	12	6	3
no use	10	4	21	12	15	5	24	8
**Endocrin** **e-therapy**								
use	12	7	18	14	20	17	19	11
no use	3	2	5	0	13	0	11	0
**Average** **age(y)**	52	46	52	53	48.	49	55	55
**Median age(y)**	55	52	47	55	50	50	55	57
**Range of** **age(y)**	29–67	26–57	34–83	31–69	28–76	37–67	32–80	38–82
**> = 50 years**	10	5	11	9	17	9	19	6
**<50years**	5	4	12	5	16	8	11	5

1 **IDC:** invasive ductal carcinoma not otherwise specified;

2 **IMPC:** invasive micropapillary breast carcinoma;

3 **ER:** estrogen receptor status;

4 **PR:** progesterone receptor status;

5 **HER2/neu:** human epidermal growth factor receptor 2 status;

6 **LVI:** Lymphovascular invasion.

### Survival comparison

The overall median follow-up duration was 51.0 months (range 5–87 months). The median follow-up was 51.0 months (range 7–87 months) in the IMPC group and 56.0 months (range 5–81 months) in the IDC group.

Multivariate analysis using Cox regression in the entire group as well as the IDC group showed that ER status was the most significant prognostic factor, followed by node status and LVI. However, the proportion of IMPC, nuclear grade and multi-LVI did not significantly correlate with survival in the IMPC group (*P*>0.05, data not shown). Node status was identified as the only independent prognostic factor in the IMPC group. The reason why the other factors didn’t show significant prognostic effect was possibly due to the limited sample size. The multivariate survival analyses by cox regression model were summarized in **Table 4**.

**Table 4:** Multivariate survival analyses by Cox regression model in all samples, IDC group and IMPC group.

	*P*-value	RR^1^	95% CI^2^	
**All samples**				
ER^3^	0.004	0.327	0.153–0.695	
LN^4^	0.004	1.709	1.190–2.453	
LV^5^	0.043	2.194	1.024–4.704	
Nuclear grade	NS^6^			
Multifocal^7^	NS			
T stage^8^	NS			
**IDC** 9				
ER^3^	0.009	0.312	0.130–0.749	
LN^4^	0.003	1.963	1.265–3.047	
LV^5^	0.041	2.467	1.037–5.870	
Nuclear grade	NS			
T stage^8^	NS			
**IMPC** 10				
LN^4^	0.018	2.236	1.151–4.343	
ER^3^	NS			
Multifocal^7^	NS			
LV^5^	NS			

1 **RR:** relative risk;

2 **CI:** confidence interval;

3 **ER:** estrogen receptor status (positive *vs* negative);

4 **LN:** lymph node status (positive lymph node *vs* negative lymph node);

5 **LV:** lymphovascular invasion (positive *vs* negative);

6 **NS:** no statistical significance;

7 **Multifocal:** primary lesion (multifocal vs solitary);

8 **T stage:** tumor size of primary lesion (T_1_ vs T_2+_).

9 **IDC:** invasive ductal carcinoma not otherwise specified;

10 **IMPC:** invasive micropapillary carcinoma.

**Figure 6:**
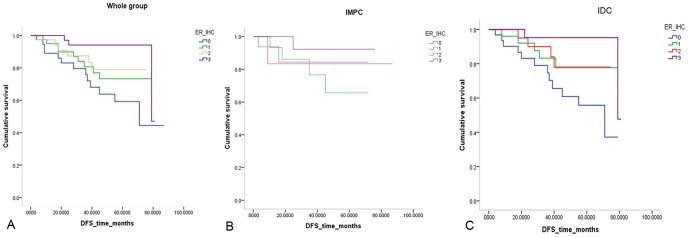
Disease free survival comparison of patients according to the semi-quantitative ER level in the whole group (*P* = .023) (A), IMPC group (*P* = .575) (B) and IDC group (*P* = .025) (C).

In all 153 cases, the semi-quantitative ER level served as a prognostic factor. The ER0 group showed the poorest prognosis, followed by the ER1+ group, and the ER3+ group displayed the best DFS (*P = *0.023). In addition, differences were also observed in the IDC group (*P = *0.025), although these trends were barely observed in the IMPC group given the small sample size (*P = *0.575) [**Figure 6A, B, C**].

**Figure 7:**
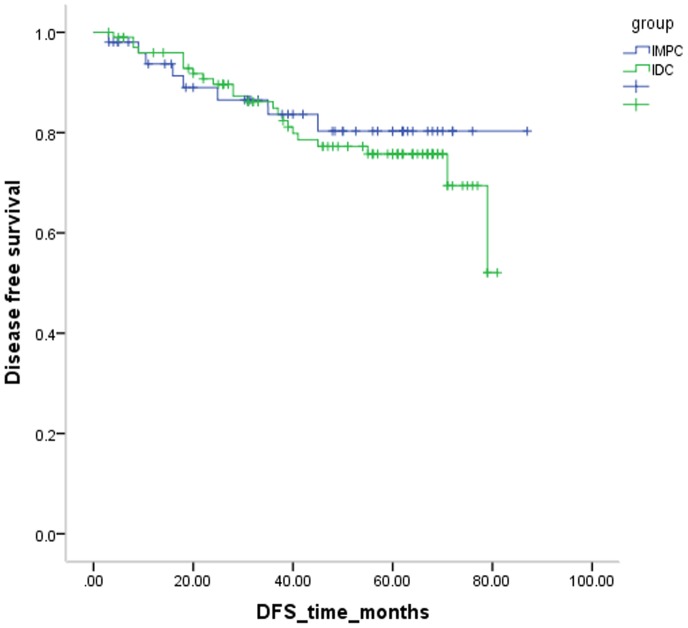
Compared with the cumulative disease free survival rate curve of invasive micropapillary carcinoma (IMPC) and invasive ductal carcinoma (IDC) (*P* = .606).

**Figure 8:**
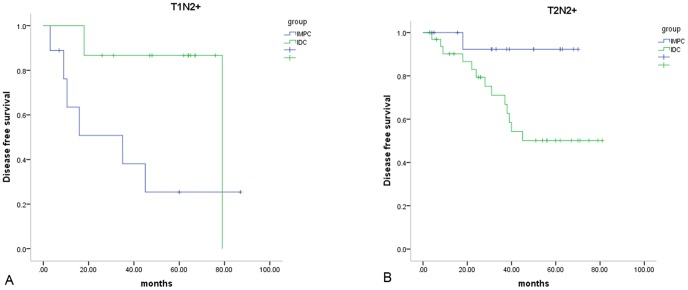
Subgroup survival comparison of invasive micropapillary carcinoma (IMPC) and invasive ductal carcinoma (IDC). Cumulative disease free survival rate curve of invasive micropapillary carcinoma (IMPC) and invasive ductal carcinoma (IDC) in T_1_N_2–3_ breast cancer (*P* = .011) (A) and T_2_N_2–3_ breast cancer (*P* = .031) (B).

In our cohort, women diagnosed with IMPC had a slightly reduced recurrence and metastasis compared to women with IDC (15.7% vs. 21.6%, *P* = 0.606) [**Figure7**]. In the subgroup analysis, IMPC patients demonstrated poorer survival (*P* = 0.011) compared to IDC patients in the T_1_N_2–3_ subpopulation, whereas IDC patients demonstrated greater recurrence and metastasis (*P* = 0.031) compared to IMPC patients in the T_2_N_2–3_ subgroup [**Figure** **8A, B**]. Then additional univariate Cox regression by bootstrapping was tested in this survival comparison. We found that IMPC patients demonstrated poorer survival compared to IDC patients in the T_1_N_2–3_ subpopulation (*P* = 0.004, RR 5.157, 95% CI 1.43–1153.28), whereas IDC patients demonstrated greater recurrence and metastasis compared to IMPC patients in the T_2_N_2–3_ subgroup (*P* = 0.045, RR 6.858, 95% CI 1.40–56.71). No difference was observed in other stage-based subgroups.

## Discussion

The reported incidence of breast cancer in China, especially in developed areas such as Shanghai, has increased significantly in recent years, and breast cancer has become one of the most common causes of cancer-related death in Chinese women [10], [11].

In a nation-wide multicenter 10-year (1999–2008) retrospective clinical epidemiological study, Li Jing et al. reported that the mean age at diagnosis in Chinese women was 48.7 years (s.d. = 10.5 years) and that breast cancer peaked in the age group from 40–49 years (38.6%), indicating that the onset of breast cancer in younger Chinese women poses a great challenge. Stanley Leong found that the peak age of breast cancer is between 40 and 50 years in Asian countries, whereas the peak age is between 60 and 70 years in Western countries. Studies have also indicated that more aggressive breast cancer characteristics are observed in young Chinese breast cancer patients. Given the above findings, we chose to match our cases according to age. The IPMC (pure or mixed with IDC) group included 51 patients ranging from 26 to 83 years of age with a median age of 51 years, and these statistics are similar to those obtained from pure IDC patients in China.

Breast cancer is a histologically heterogeneous disease that displays various biological behaviors and pathologic subtypes [12]. IMPC is a rare histologic type that appears to be more aggressive compared to other breast cancers [13], [14] due to its increased probability of lymphovascular invasion and LN metastasis. IMPC typically demonstrates a LN metastasis rate of 46–95% [15], [16], [17], [18] compared with 34% for IDC. Therefore, given IMPC’s highly lymphotropic nature at initial presentation, IMPC has an unfavorable prognosis compared to IDC and therefore merits aggressive treatment. In this study, we both included pure and mixed IMPC cases because of the low incidence of pure IMPC. In Caterina Marchio’s article, it was pointed out that mixed IMPCs harbored similar patterns of genomic aberrations and phenotype compared to pure IMPCs. And they proved that mixed IMPCs were more closely related to pure IMPCs than to IDC-NSTs [19]. Very few studies have compared the prognosis of IMPC and IDC between patients matched for node status and age group at initial diagnosis. However, previous studies have shown that node status is the strongest predictor of DFS and overall survival (OS) [20]. Jeong Il Yu et al [21] conducted a case-control study in a Korean population comparing IMPC and IDC patients with the same TNM stage, and their study showed that the recurrence-free survival (RFS) and 5-year local regional RFS (LRRFS) rates of IMPC patients were significantly higher in IMPC patients compared to IDC patients, whereas no difference in OS was observed. These results are inconsistent with our results, which can likely be attributed to differences in rates of ER positivity. In our target population, the ER-positive rate in the IPMC group was 84.3%, which was significantly higher than that in the IDC group (68.6%, *P* = 0.037). However, the ER-positive rate reported in J.I. Yu’s paper was 75% for the IMPC group and 73.6% for the IDC group. These previous authors defined an Allred score greater than 3 as ER positive, and this standard was stricter than our criterion of >1% positive nuclei. However, we used the same criteria for the IDC and IMPC group, and our IMPC group demonstrated an increased ER-positive rate. This difference represents one potential explanation for these survival results.

In previously reported IMPC papers, ER positivity ranges between 25% and 91%, but most of these data were derived from studies with small sample sizes. Albert C. Chen et al [18] conducted a population-based study using data from the National Cancer Institute’s Surveillance, Epidemiology and End Results (SEER) database, and their study of 624 IMPC patients represents the largest retrospective IMPC data set to date. These authors reported an ER positivity rate of 85%, which is consistent with our study. However, one limitation of the SEER database is that data on pathological characteristics, such as LVI, multifocal status and adjuvant therapies, were not included.

The NSABP-B14 study [22] indicated that low levels of ESR1 mRNA may represent a mechanism for tamoxifen resistance. However, our study included more ER-positive patients in the IMPC group, which indicates that the primary endpoint between 2 groups would be mixed.

Although node status was matched between these 2 groups, LVI positivity remained significantly higher in IMPC patients (52.9%) compared to IDC patients (29.4%, *P* = 0.007). However, this difference was not associated with worse prognosis for IMPC patients compared to IDC patients in our study, although LVI and LN status are typically identified as prognostic factors. This finding can be partially attributed to the fact that IMPC patients considered to have a poorer prognosis according to the physician were more likely to receive chemotherapy than IDC patients. Moreover, this trend was not statistically significant, with the exception of anthracyclines, which were administered to 92.1% of IMPC patients and 79.4% of IDC patients (*P* = 0.044).

Traditionally, increased tumor size and LN involvement have been considered independent predictors of recurrence and metastasis. The research of Jennifer Y. Wo demonstrated that very small tumors with 4 or more positive LNs are predictive of increased breast cancer-specific mortality [23]. To more effectively compare IMPC and IDC patients, we sought to characterize the interaction between tumor size and LN involvement in DFS. For this study, patients were classified into the following 4 subgroups: T_1_N_0–1_, T_1_N_2–3_, T_1_N_2–3_ and T_2–3_N_2–3_.

In the subgroup analysis, the T_1_N_2–3_ subgroup was more likely to be associated with poor prognosis for the IMPC group. However, the T_2–3_N_2–3_ subgroup demonstrated the highest rate of recurrence and metastasis in the IDC group. Thus, the biological behavior of IMPC and IDC groups is different; the IDC group follows the trend whereby larger primary lesions have increased local LN involvement [22], whereas the IMPC group demonstrates more common invasion of the lymphatic system.

Hence, we compared DFS among IDC and IMPC patients in the 4 subgroups. We concluded that IMPC patients had significantly poorer survival (*P* = 0.018) compared to IDC patients in the T_1_N_2–3_ subpopulation, whereas IDC patients had significantly more recurrence and metastasis (*P* = 0.024) compared to IMPC patients in T_2_N_2–3_ subgroup. With chi-square by bootstrapping, no significant difference was found in chemotherapy between IMPC and IDC in T_1_N_2–3_ subgroup (*P* = 0.290).

The conventional view of metastasis is that cancer gains metastatic ability as tumors grow larger through an accumulation of mutations, and this notion was supported by the results in IDC patients but not IMPC patients. We hypothesize that smaller IMPC with extensive nodal spread might represent a specific subset, which need further validation.

We randomly chose six mixed IMPC and their paired fresh samples of IDC from our tissue bank and used multiplex bDNA assay [24] to compare the mRNA expression of IMPC and IDC groups. Then we defined absolute fold change ≥2.0 as significant difference between two groups and found out that ESR1, GSTM1, MAOB, KRT5 et al had differential expression between the groups [**Data not shown**]. After we build up the system which could use formalin fixed paraffin embedded samples to analyze, more data would be provided.

### Limitations

Further analysis of more patients with longer follow-up is needed to verify the prognosis of IMPC in Chinese women. Selection bias was not completely avoided because this was a retrospective cohort study. More specifically, as to chemotherapy, We observed a significantly higher proportion of patients receiving a regimen containing anthracycline in the IMPC group compared to the IDC group, which might interfere with analysis. Another limitation of this study was that Ki-67 pathological data were not routinely obtained from patients, while Ki-67 was commonly used as a prognostic factor.

### Conclusion

Our study demonstrated that the prognoses of IMPC and IDC patients matched for LN status and age were comparable. Therefore, the poorer prognosis of IMPC patients may be attributed to an increased probability of nodal involvement at initial diagnosis, rather than other intrinsic biological characteristics. Moreover, IMPC patients demonstrated a significantly poorer outcome compared to IDC patients with smaller tumors and 4 or more positive LNs. The opposite result was observed in larger tumors with 4 or more positive LNs. If this conclusion were validated by research using a larger sample size, we might recommend more proactive treatment for IMPC patients with a smaller tumor size and extensive LN involvement. Next, we plan to assess the gene expression profile of archival formalin-fixed IMPC and IDC samples, especially those with small primary lesions and high LN involvement. In addition, we will explore tumor markers, such as ESR1, and use more samples to validate and identify prognostic factors.
